# Information and Scientific Impact of Advanced Therapies in the Age of Mass Media: Altmetrics-Based Analysis of Tissue Engineering

**DOI:** 10.2196/25394

**Published:** 2021-11-26

**Authors:** Antonio Santisteban-Espejo, Miguel Angel Martin-Piedra, Antonio Campos, Julia Moran-Sanchez, Manuel J Cobo, Ana I Pacheco-Serrano, Jose A Moral-Munoz

**Affiliations:** 1 Department of Pathology Puerta del Mar University Hospital Cadiz Spain; 2 Institute of Research and Innovation in Biomedical Sciences of the Province of Cadiz (INiBICA) University of Cadiz Cadiz Spain; 3 Department of Histology Tissue Engineering Group University of Granada Granada Spain; 4 Department of Medicine University of Cadiz Cadiz Spain; 5 Department of Hematology and Hemotherapy Puerta del Mar University Hospital Cadiz Spain; 6 Department of Computer Science and Engineering University of Cadiz Cadiz Spain; 7 Department of Nursing and Physiotherapy University of Cadiz Cadiz Spain

**Keywords:** advanced therapies, tissue engineering, scientometrics, altmetrics, online, web, communication of science

## Abstract

**Background:**

Tissue engineering (TE) constitutes a multidisciplinary field aiming to construct artificial tissues to regenerate end-stage organs. Its development has taken place since the last decade of the 20th century, entailing a clinical revolution. TE research groups have worked and shared relevant information in the mass media era. Thus, it would be interesting to study the online dimension of TE research and to compare it with traditional measures of scientific impact.

**Objective:**

The objective of this study was to evaluate the online dimension of TE documents from 2012 to 2018 using metadata obtained from the Web of Science (WoS) and Altmetric and to develop a prediction equation for the impact of TE documents from altmetric scores.

**Methods:**

We analyzed 10,112 TE documents through descriptive and statistical methods. First, the TE temporal evolution was exposed for WoS and 15 online platforms (news, blogs, policy, Twitter, patents, peer review, Weibo, Facebook, Wikipedia, Google, Reddit, F1000, Q&A, video, and Mendeley Readers). The 10 most cited TE original articles were ranked according to the normalized WoS citations and the normalized Altmetric Attention Score. Second, to better comprehend the TE online framework, correlation and factor analyses were performed based on the suitable results previously obtained for the Bartlett sphericity and Kaiser–Meyer–Olkin tests. Finally, the linear regression model was applied to elucidate the relation between academics and online media and to construct a prediction equation for TE from altmetrics data.

**Results:**

TE dynamic shows an upward trend in WoS citations, Twitter, Mendeley Readers, and Altmetric Scores. However, WoS and Altmetric rankings for the most cited documents clearly differ. When compared, the best correlation results were obtained for Mendeley Readers and WoS (ρ=0.71). In addition, the factor analysis identified 6 factors that could explain the previously observed differences between academic institutions and the online platforms evaluated. At this point, the mathematical model constructed is able to predict and explain more than 40% of TE WoS citations from Altmetric scores.

**Conclusions:**

Scientific information related to the construction of bioartificial tissues increasingly reaches society through different online media. Because the focus of TE research importantly differs when the academic institutions and online platforms are compared, basic and clinical research groups, academic institutions, and health politicians should make a coordinated effort toward the design and implementation of adequate strategies for information diffusion and population health education.

## Introduction

Tissue engineering (TE) is a multidisciplinary field aiming to develop biological substitutes that can restore, maintain, or even improve the structure or functionality of damaged tissues [1]. Since its appearance in 1988 [2], TE has globally spread to improve current therapeutic approaches, entailing a revolution in health sciences [3]. In this sense, several TE devices have been employed in the treatment of damaged blood vessels [4], peripheral nerve injuries [5], chronic skin ulcerations [6], oral mucosal replacement [7,8] and corneal lesions [9].

The crescent interest and the fast development of TE have been demonstrated from a quantitative perspective by showing the incremental number of TE publications during the last decade [10]. Moreover, its cognitive and social frameworks have been described by means of science mapping analysis techniques [11]. These bibliometric-based studies can serve as a guide to help administrative authorities to better plan funding allocations and to promote synergies among research groups, as previously exhibited in other scientific areas [12,13].

In this sense, traditional bibliometric analysis employs the information extracted from academic documents (ie, citations or keywords) to comprehend the evolution of a scientific discipline, such as TE [10,11,14]. However, classical bibliometric methods have been largely reviewed because of their fewer adequacy to assess the real dimension of scientific enterprise and due to a relative inattention to the societal dimension of scientific endeavor [15]. Consequently, a new kind of metrics, called alternative metrics or altmetrics, has been proposed to obtain, evaluate, and characterize scientific information through data content in social media [16].

Altmetrics describes a web-based metrics used to understand the impact of publications and other scholarly materials by using data from social media platforms (ie, Twitter, Facebook, Google+, blogs, Mendeley Readers, CiteULike, Reddit, and Wikipedia, among others) [17]. The emergence and development of these metrics are related to the social media revolution: there are now different groups of the population, nonauthor professionals, which read research articles and also share them; furthermore, new types of academic outputs have appeared [18]. Hence, the traditional acceptance that scientific output is disseminated solely through academic media, such as journals, conferences, or specialized books, has now changed.

In addition, the online public nature of these metrics allows to track mentions of scholarly articles across the online landscape faster and broader than traditional citation metrics [19]. The validity and potential of altmetrics and its necessary collaborative relation with classical metrics have been demonstrated in several disciplines [20]. Motivations on the impact that these metrics could offer on professional research careers have been also scrutinized [21].

Then, within the context of a global science where information is shared and consumed in the web, even before its general validation for the scientific community, it would be interesting to explore the online dimension of a multidisciplinary and dynamic science such as TE. Among the recent advances in health sciences, the construction of biosimilar tissues constitutes one of the most powerful approaches to achieve the successful treatment of previously untreated conditions. To the best of our knowledge, there are no documents available that evaluate the online dimension of TE research since its appearance at the end of the 20th century. Thus, the primary aim of this study was to determine the characters of TE behavior online and to compare it with traditional metrics of scientific impact.

## Methods

### Sample

The metadata used in this study were obtained from the Web of Science (WoS) Core Collection bibliographic database. WoS is considered one of the most relevant scientific information sources, as it contains reliable evidence about citations, and is widely used in research evaluations [22].

The search strategy used in this study was “TISSUE ENGINEER*” or “TISSUE-ENGINEER*”, and it was applied on the Science Citation Index-Expanded Collection for a period between 2012 and 2018. We performed this search strategy to accurately discriminate between genuine TE documents and documents belonging to other related areas such as regenerative medicine or cellular therapy [23]. As originally described by Langer and Vacanti [1], TE is defined by the use of cell sources, matrices, and growing factors to construct biomimetic tissues with a therapeutic impact on human health [1], which differs from other emerging biomedical approaches based on the sole use of cultured stem cells or biomaterials without giving rise to a human bioartificial tissue. In this sense, our aim was to capture this precise notion of TE research.

Once the metadata were extracted, we excluded reviews, book chapters, meeting abstracts, and proceeding articles. Then, original articles obtained from this research were matched with the information available on Altmetric online [24], which holds important social information since 2012 from a much broader spectrum of sources than traditional metrics (eg, web-based references, news media mentions, Twitter mentions, or patents, among others) [25].

### Descriptive Analysis

To comprehend the behavior of TE in the social web and to compare it with traditional metrics, we carried out 2 different analyses. First, we evaluated the presence of original articles regarding TE in 7 different platforms (WoS, Altmetric Attention Score, Twitter, patents, Facebook, Mendeley Readers, and news) as the percentage of documents with at least one mention or a citation from 2012 to 2018. Following Eysenbach [26], in the case of Twitter, we called each mention a *tweetation*, which includes the mention of a TE journal article URL, retweet of the same tweet, or sending a modified tweet by other users [26]. In addition, we obtained the top 10 most cited TE original articles from 2012 to 2018 and ranked them according to 2 parameters: the normalized WoS citations and the normalized Altmetric Attention Score. Those measures were calculated using the rationale of the normalized citation impact. It was calculated by dividing the count of citing items by the average of citations for documents with the same year of publication in our corpus of documents. The Altmetric Attention Score has been previously employed as a bibliometric measure of online attention [25].

### Statistical Analysis

To better characterize TE structure online, we performed 3 different statistical tests: Spearman correlation test [27], factor analysis [28], and linear regression model [29]. The collection of cites using traditional metrics requires several years, while the data provided by Altmetric before 2015 were not extensive, as the platform was only founded in 2012. For this reason, correlation and factor analyses were performed on publications retrieved from 2015 to 2018. This strategy has been used previously in other altmetrics studies [30]. Furthermore, all citation and mention counts were transformed with the formula Ln(1+x) before processing to reduce skewing [30].

To verify that the data set does not follow a normal distribution, the Kolmogorov–Smirnov test was performed for the next 16 variables that were evaluated, overall, to characterize the field: (1) WoS citations, (2) news, (3) blogs, (4) policy, (5) Twitter, (6) patents, (7) peer review, (8) Weibo, (9) Facebook, (10) Wikipedia, (11) Google, (12) Reddit, (13) F1000, (14) Q&A, (15) video, and (16) Mendeley Readers. The Spearman correlation was then obtained for the variables previously described, and the statistical significance was defined as *P*<.05.

Once the correlation data were obtained, factor analysis was performed. Factor analysis allowed us to identify the common variables or factors that could explain the previously observed correlation data. In this sense, Bartlett sphericity and Kaiser–Meyer–Olkin tests were performed prior to assessing the suitability of factor analysis [31]. Finally, the linear regression model was applied to obtain a mathematical expression of the influence of alternative metrics on a traditional measure of scientific impact such as the citation counts. The equation constructed contains a group of variables identified in the correlation and factor analyses, which allows us to predict the number of WoS citations in 2018 from 2015 TE Altmetric scores. Finally, the equation was used to calculate the predicted citation(s) of the documents published in 2015. A *t*-test analysis was employed to determine the significance (95% CI and significance at *P*<.05). JASP (freeware; University of Amsterdam, Amsterdam, The Netherlands) was employed to perform all the statistical analyses [32].

## Results

### Sample

After performing the search strategy described, a total of 23,179 documents pertaining to TE were retrieved from WoS for the period from 2012 to 2018. A process of matching between the DOIs available in the WoS and the Altmetric data was then performed. Finally, a total of 10,112 documents (43.63%) with an Altmetric score of 1 or higher were obtained.

### Descriptive Analysis

#### Evolution of TE Documents in WoS and the Online Web

**Figure 1:**
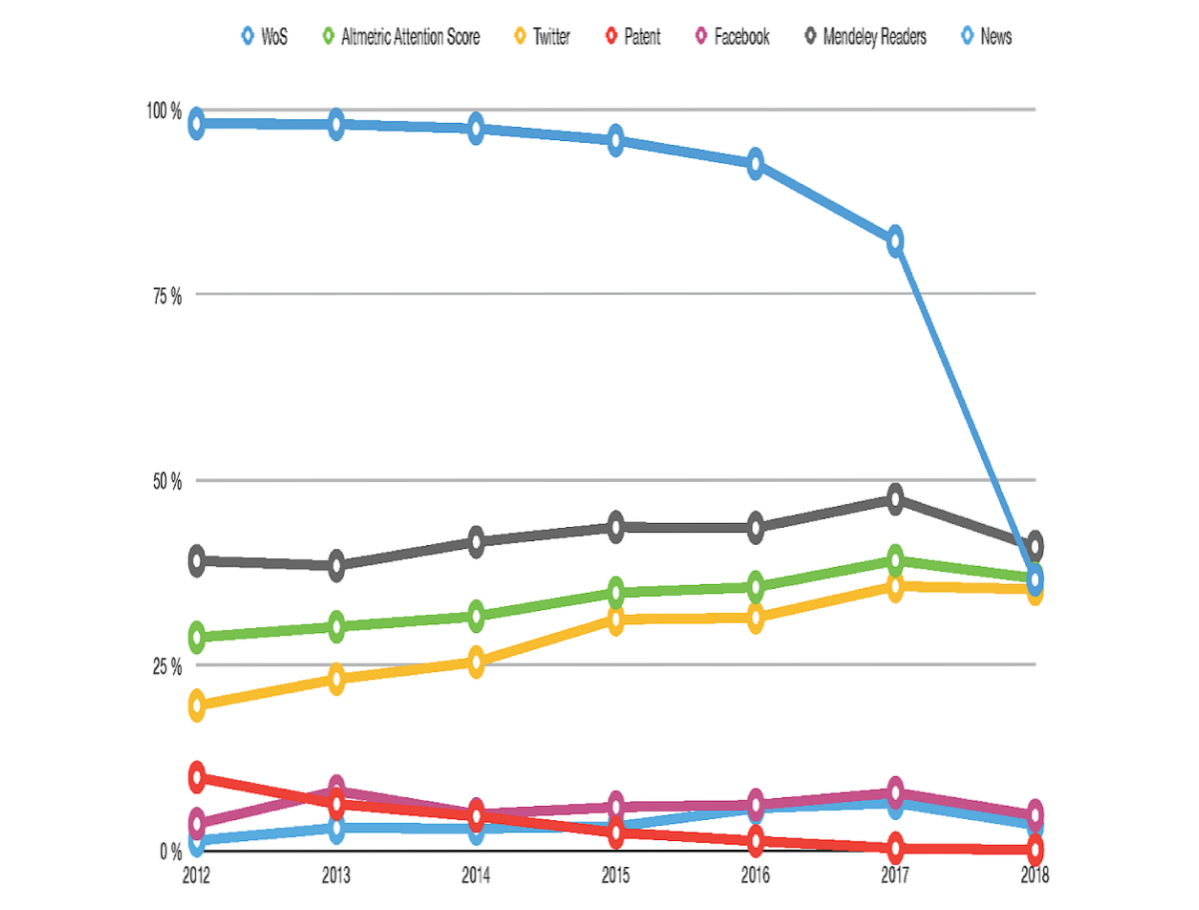
Percentage of documents with at least one citation/mention for the period 2012-2018. Only those platforms with more than 5% in any year were represented. WoS: Web of Science.

The presence of TE documents in WoS and online is shown in Figure 1. The trend lines indicate the evolution of the percentage of documents with at least one citation or mention during the period 2012-2018. In WoS, the percentage of documents exceeds 85.00% from 2012 to 2017. However, the nearness of 2018 to the time of data acquisition explains the result of WoS citations in that year (40.93%) when these metadata were not already collected. The evolution of TE documents in Twitter, Mendeley Readers, and the Altmetric Attention Score shows an upward trend from the beginning of the period studied. In this sense, documents with at least one mention in the reference manager Mendeley Readers were close to the 50% in 2017. By contrast, the presence of TE documents in platforms such as Facebook, patents, and news was less than 10% for the whole period studied.

#### Ranking of TE Documents According to WoS Citations and Altmetric Attention Score

The top 10 TE documents ranked by their normalized WoS citations and normalized Altmetric Attention Score are presented in Tables 1 and 2.

**Table 1:** Top 10 tissue engineering documents ranked by WoS^a^ citations for the period 2012-2018.

WoS rank	Altimetric rank	Normalized WoS citations	Normalized Altmetric Attention Score	Reference
1	5	38.32	140.76	[33]
2	21	35.19	54.11	[34]
3	43	22.19	33.31	[35]
4	602	21.49	2.16	[36]
5	291	19.78	5.73	[37]
6	6022	18.10	0.11	[38]
7	8060	18.10	0	[39]
8	2259	18.03	0.59	[40]
9	3738	16.97	0.22	[41]
10	8	14.91	68.29	[42]

^a^WoS: Web of Science.

**Table 2:** Top 10 tissue engineering documents ranked by Altmetric Attention Score for the period 2012-2018.

WoS^a^ rank	Altimetric rank	Normalized WoS citations	Normalized Altmetric Attention Score	Reference
365	1	3.46	204.96	[43]
16	2	12.97	199.36	[44]
83	3	6.83	149.21	[45]
39	4	9.60	141.62	[46]
1	5	38.32	140.76	[33]
8853	6	0	115.27	[47]
1584	7	1.73	85.10	[48]
10	8	14.91	68.29	[49]
307	9	3.80	67.04	[50]
252	10	4.37	65.81	[51]

^a^WoS: Web of Science.

Tables 1 and 2 show a remarkable discrepancy between classical (normalized WoS citations) and alternative (normalized Altmetric Attention Score) metrics among the most valued documents.

On the one hand, the original article by Deng et al [39], reporting multifunctional stimuli-responsive hydrogels with self-healing, high conductivity, and rapid recovery through host–guest interactions, has a remarkable scholarly impact, being the 7th top-cited document when analyzing normalized WoS citations. However, the Altmetric Attention Score was null for this paper, suggesting that in vitro research could not attract as much societal attention as translational research. On the other hand, the research study by Nichols et al [47], regarding the transplantation of bioengineered lung into a large-animal model, employs a very translational approach to TE, and thus its social impact is reflected by the high Altmetric Attention Score, although its scholar relevance was not yet evident.

### Statistical Analysis

#### Correlation Analysis

The results of the correlation analysis between traditional and alternative metrics of all retrieved publications from 2015 to 2018 are presented in Table 3.

**Table 3:** Spearman correlation results between pairs of variables for tissue engineering articles published from 2015 to 2018.^a^

	WoS^b^	News	Blogs	Policy	Twitter	Patents	Peer review	Weibo	Facebook	Wikipedia	Google	Reddit	F1000	Q&A	Video
WoS															
News	*0.144*														
Blogs	*0.137*	*0.387*													
Policy	0.049	0.065	0.064												
Twitter	*0.176*	*0.149*	*0.158*	0.009											
Patents	*0.114*	*0.093*	*0.09*	–0.009	–0.068										
Peer review	–0.006	–0.01	*0.136*	–0.001	–0.006	–0.009									
Weibo	0.04	*0.108*	*0.108*	–0.002	0.063	–0.011	–0.002								
Facebook	0.064	*0.2*	*0.213*	0.046	*0.213*	0.047	–0.014	*0.081*							
Wikipedia	0.076	0.072	0.073	–0.004	0.073	0.032	–0.004	*0.14*	0.039						
Google	0.071	*0.109*	*0.184*	*0.138*	0.073	0.013	–0.005	*0.11*	*0.184*	0.086					
Reddit	–0.012	0.008	0.028	–0.005	–0.015	–0.03	–0.005	*0.118*	0.016	–0.013	0.025				
F1000	0.054	0.036	0.063	–0.003	0.05	0.013	–0.003	*0.164*	0.015	0.063	0.046	–0.011			
Q&A	0.003	–0.007	–0.007	–0.001	–0.004	–0.006	–0.001	–0.001	–0.01	–0.003	–0.003	–0.003	–0.002		
Video	–0.033	0.008	0.036	–0.003	0.006	–0.02	–0.003	–0.004	0.061	–0.009	–0.011	–0.011	–0.008	–0.002	
Mendeley Readers	*0.716*	*0.198*	*0.197*	0.021	*0.243*	*0.104*	–0.025	0.049	*0.107*	0.077	*0.098*	0.012	0.067	0.008	0.03

^a^Italicized values mean *P*<.05.

^b^WoS: Web of Science.

Overall, the number of citations on Mendeley Readers and WoS shows the best correlation results (ρ=0.71) and platforms such as Twitter (ρ=0.17) and news (ρ=0.14) have a suitable correlation. However, the correlation results obtained for Wikipedia, Facebook, F1000 mentions, and Q&A mentions were weak, and an inverse correlation was observed for TE documents appearing in 3 online platforms: peer review mentions (ρ=−0.006), Reddit mentions (ρ=−0.01), and video mentions (ρ=−0.03).

#### Factor Analysis

**Figure 2:**
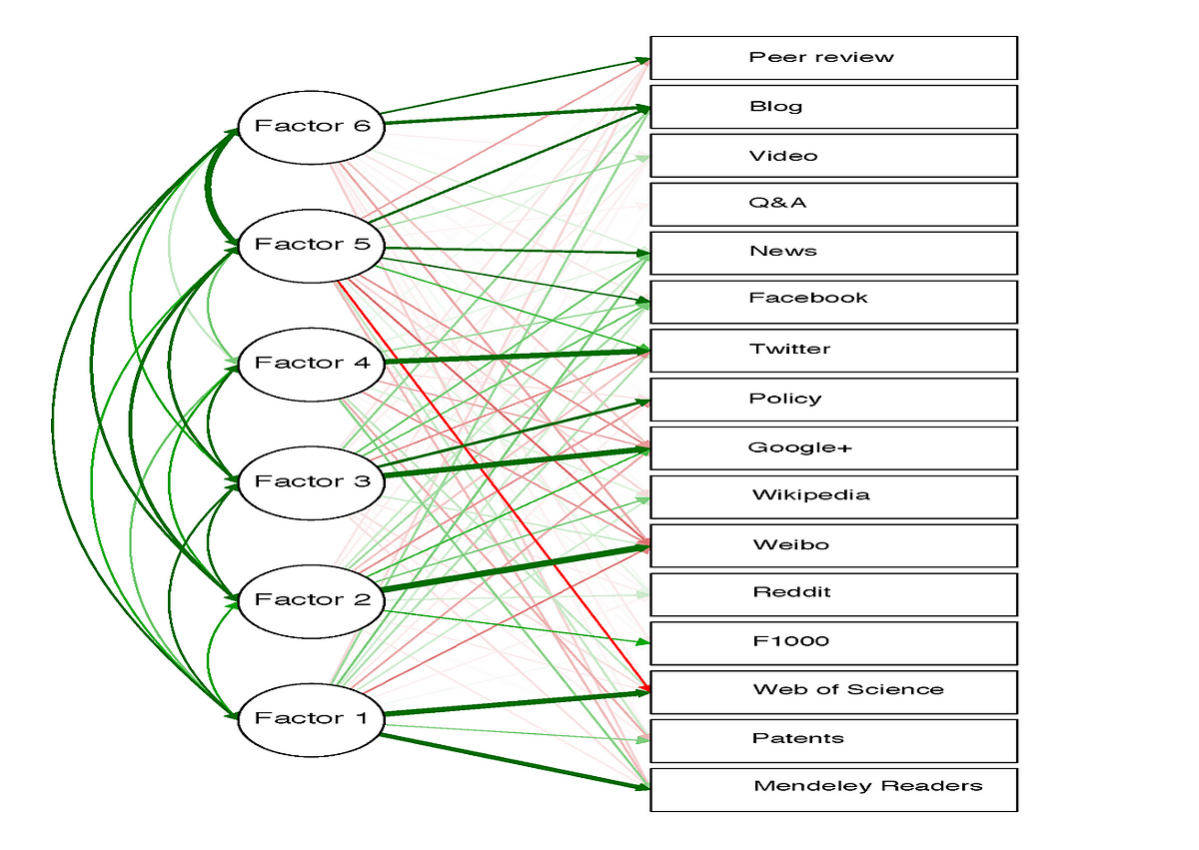
Results of the factor analysis for the 2015 production in the research field of tissue engineering.

First, a value of 5629.85 (*P*<.001) for a chi-square approximation of the Bartlett sphericity test and a value of 0.700 for the Kaiser–Meyer–Olkin test confirmed the suitability of factor analysis. Then, factor analysis identified 6 different components or factors that could explain the correlation results. These factors are shown in Figure 2 and labeled as F1, F2, F3, F4, F5, and F6. Positive and negative results are indicated with green and red lines, respectively.

F1 exposes the relation between WoS citations and Mendeley Readers. However, the remaining factors (F2-F6) most likely account for a different type of scientific impact not directly associated with TE professional researchers and readers. Regarding this, F3 acts as a common factor for Google and policy mentions, and, interestingly, 3 social platforms (blogs, news, and Facebook) appear together within F5. Finally, the mentions of TE documents in Twitter (*tweetations*) were strongly tied with a unique factor (F4), suggesting a particular behavior and structure for TE information shared in Twitter.

#### Linear Regression Analysis

The correlation coefficient (r) and the determination coefficient (R^2^) obtained were equal to 0.645 and 0.414, respectively. In addition, the statistical test for the analysis of variance was significant (*P*<.001). Consequently, the mathematical model constructed explains more than the 40% of the variation in the number of WoS citations obtained for TE documents in 2018 from 2015 Altmetric scores.

The variable Mendeley Readers constitutes the best citation predictor for TE documents as it holds the higher result for r (r=0.599). The rest of the Altmetric scores also had a positive correlation but the strength of the observed association was weaker.

The prediction equation for 2018 TE WoS citation counts from 2015 Altmetric scores can be expressed as follows:

Ln (1 + WoS)=–27.25 + 5.37 x Ln (1 + blog) + 0.82 × Ln (1 + news) + 12.78 × Ln (1 + Mendeley Readers) + 5.83 × Ln (1 + patent) + 0.75 × Ln (1 + Twitter).

Finally, no significant differences were found (*P*=.12) for the predicted citations versus the real citations rates for the documents published in 2015.

## Discussion

### Principal Findings

The seminal article published by Langer and Vacanti [1] laid the foundations for TE. Since then, TE has evolved and given rise to an interdisciplinary field that applies the principles of engineering and life sciences toward the development of biological substitutes that can restore, maintain, or even improve tissue functions. Within contemporary medicine, TE is considered one of the most promising advanced therapies, as it has the potential to overcome traditional problems associated with organ failure and to treat previously untreated conditions [52]. Its onset and application to the clinical practice have led to a revolution in surgery and transplantation procedures, as new bioartificial tissue devices are now available for therapy with a considerably less risk of infection transmission and immune-mediated organ rejection [53].

As a consequence, a crescent interest has appeared, aiming to elucidate global trends in TE and its cognitive and social framework [10,11]. These bibliometrics-based approaches utilize, in common, the traditional measures of scientific impact, such as citations and publications. Moreover, the social maps and conceptual diagrams proposed suffer from the same bias, as both the relations among institutions and the key notions identified are based on the number of citations and co-occurrence of keywords [54]. Thus, new approaches are needed to better characterize and comprehend the real impact of TE in our society. In this sense, the association of traditional bibliometrics with alternative metrics (altmetrics) could render a more sensible and realistic view of TE behavior nowadays [55].

Hence, in this study, we have carried out an altmetrics-based analysis of the core documents of TE retrieved from WoS between 2012 and 2018. We have previously employed this query term to analyze the global trends of TE [10], the cognitive and social framework of TE [11], and the structure and evolution of TE reviews [56], in an attempt to replicate the same search strategy highlighting the value of the reproducibility and comparability of our results. To our best knowledge, there is no previous literature that defines TE structure and its major characters online or its essential divergence with other widespread platforms in clinical medicine such as scientific journals.

In this regard, we first performed a descriptive analysis of evolution of TE documents in WoS and 6 different web-based platforms (ie, Facebook, patents, Twitter, news, Mendeley Readers, and Altmetric Attention Score). The presence of TE documents in WoS is significant over the rest, suggesting the existence of a well-established research dynamic where academic and professional health practitioners collect and consult applicable clinical information in renowned databases. Besides, TE diffusion in Twitter stood out within the group of social networks consulted; it is interesting to note a growing trend for the whole period evaluated, and a particular pattern of scientific information diffusion in Twitter could explain these results.

On the one hand, the own structure of Twitter, a micro-blogging platform that enables the users to “tweet” short messages with their virtual colleagues, has developed a singular model of scientific communication and a special information flow [57,58]. Kwak et al [58] demonstrated that retweets constitute the nucleus of this original model. Hence, retweets of TE documents could spread their information beyond the limits of their original authors, expanding them to the broad space of the followers’ networks [59,60]. In addition, relevant information about new TE devices may reach primary care physicians and groups of patients through this network, optimizing the communication between different health care levels and the education of society [61]. Eysenbach [26] reported that highly tweeted articles are 11 times more likely to end up as being highly cited and that Tweets correlate with traditional metrics of scientific impact [26]. Consequently, the upward trend of TE documents in Twitter could also be explained in terms of this higher academic impact.

To better comprehend the similarities and differences between the focus of TE documents online and in traditional scholar media, we identified the 10 most cited TE documents from 2012 to 2018. We then ranked and compared them according to the number of normalized WoS citations and the normalized Altmetric Attention Score. The results obtained demonstrated a clear discrepancy between the rankings of TE documents, suggesting that citations in WoS and interests of online users do not follow the same path. Differences between metrics tend to be more remarkable when comparing the top-ranked documents for each metric. This comparison, although cannot be used for validation purposes, is useful to elucidate this differential pattern. This kind of dissimilar relation, where scholar- and web-based attention clearly differs, has never been demonstrated for TE as a discipline, although it is not exclusive of it. In this sense, similar results have been shown in other research fields, revealing that social and academic assumptions of scientific advances are not guided by identical principia [62,63].

This finding is not a negative result but rather a consequence of the varying nature of traditional and alternative metrics, as well as the social and dynamic context in which research takes place. It has been reported that, in medical and applied sciences, an important share of information targets is found outside the research and scholar community and that traditional citations are only partial measures of impact and use of information [64]. In accordance with Bornmann [65], citations only assess the impact of scholarly literature on those who cite, and this neglects many audiences of scholarly literature who may read the paper, but do not cite it as “pure” readers [65]. Furthermore, the task of assessing the impact of science has to take into account some policy and society demands. These societal, policy-driven, and technical demands have led to the emergence of altmetrics as an evolved methodology to broaden the impact of research on both researchers and policy demands as promoters of research and society as final users of developed technology through advances in research.

As TE is devoted to the construction of biomimetic tissues that can restore, maintain, or even improve the structure or functionality of damaged tissues [3], and to treat previously untreated conditions [66,67], its social demands are particularly important [68]. In this sense, the use of altmetrics, combined with classical measures of scientific impact, could provide a wider context on the real influence of TE research in society.

In addition to the descriptive analysis, we applied 3 different statistical tests: Spearman correlation, factor analysis, and the linear regression model. The correlation study showed that TE citations in WoS and the number of readers in Mendeley Readers have the highest value (ρ=0.71). This finding can be explained by attending to the own nature of Mendeley Readers, as it is a citation manager tool essentially used to store and share references by a community of bibliographic users. The use of Mendeley Readers has been previously correlated with future citation counts in several biomedical sciences fields [69]. In this way, citations of TE documents in WoS are equally well-correlated with the number of Mendeley Readers. Because TE researchers could use the previously stored documents as cited documents for their own future publications, the correlation results are, to some extent, explainable. However, Mendeley Readers users do not have to be publishing academics exclusively, and may also be practitioners or students, as previously demonstrated [70,71]. Therefore, the correlation observed in TE research should be related to a broader spectrum of scientific activity and not just restricted to experts and research groups that publish in specialized journals.

A positive but weaker correlation was obtained for online platforms such as Twitter, news, and blogs. However, the mention of TE documents in video and Reddit is lesser, because of an inverse correlation. These results are most likely influenced by the structure and the type of readers on these platforms. For example, in Reddit, virality constitutes a crucial factor [72]. As stated by Berger and Milkman [73], those contents that evoke emotions of activation (eg, anger, awe, anxiety) are more suitable to become viral, in contrast to deactivating emotions (eg, softness) [73]. Hence, documents referring to the construction of bioartificial tissues could be mentioned in Reddit to be criticized or report findings that are surprising and shocking for common readers, but not so relevant for a specialized audience.

For instance, correlation studies could obscure the genuine relationships existing between a set of variables. This potential bias is particularly important when a predominant or strong association exists [30]. In this sense, factor analysis could serve to identify the common factors or components that explain the previously observed correlation. In this regard, factor analysis of TE production showed the existence of 6 differentiated factors (F1-F6).

F1 is tied to readers in Mendeley Readers, citations in WoS, and patents. Interestingly, the final goal that guides TE research is the clinical application of bioengineered tissue devices in the daily practice of the medical specialties. For this achievement, 2 previous requirements must be guaranteed: the communication of the scientific results in a peer-reviewed journal and the acquisition of a patent license. As this process is causally related to the employment of citation manager and paper collection in well-known databases, factor analysis reveals consistent results. F2 (Weibo, F100, Reddit, and Wikipedia) probably accounts for a different kind of TE information consumption. A more informal communication of results with less scientific rigor mostly presided over the components that integrate this factor.

The relation between policy and Goggle in F3 is not clear, as the latter can be used to filter and obtain a heterogeneous and vast amount of information related to TE, and not just the legal requirements for TE application in clinics. It is interesting to note that Twitter acquires an individual dimension in F4, constituting a social network distinguished from the rest. Nevertheless, news and Facebook appear together in F5 and blogs constitute a component of F6. A plausible explanation for this leading role of Twitter in the diffusion of TE information is that the development of TE has taken place in parallel with the burst of social media. Probably, as previously stated for other scientific disciplines, TE researchers have substituted the idea of academic community for the virtual department [74,75]. Moreover, the structural multidisciplinary nature of TE and the relationships between the biomaterials industry, research groups, and clinicians can be ideally displayed using a social network such as Twitter [10,58,76].

Finally, we aimed to develop a mathematical model for TE documents to predict the influence of Altmetric scores on future citation counts with relative accuracy. However, in accordance with Thelwall and Nevill [30], it is reasonable to consider alternative metrics in conjunction with journal impact to get an idea about which articles are more likely to attract longer-term citations [30]. Applying this logic to TE production, we established a linear regression equation to derive 2018 TE citation counts from 2015 Altmetric indexes. The model is able to explain more than the 40% of variation in the number of WoS citations for TE documents that Altmetric tracked (R^2^=49.6%); regression results were statistically significant, and so the association between measures such as publications or citations and the impact of scientific work online could serve to better characterize the movement of information in biomedical disciplines, such as TE.

We hope this article serves to stimulate the adequate use of web-based platforms in the communication and diffusion of scientific information in TE. We are firmly convinced that, as wisely stated by Weigold [77], the sharing of well-constructed information online contributes to informing society about real possibilities of scientific progress.

### Limitations

Although the findings provided in this study are interesting, several limitations must be addressed. First, only a percentage of the publications indexed in WoS are available on Altmetric, and consequently, the conclusions of the study are influenced by the core of documents obtained. Second, the factor analysis is performed for only 1 year; although the behavior of the research area could be similar, it could be influenced by the published topics or other factors. Finally, the intentional tweeting by the publisher or the editor of the journal was not analyzed.

Furthermore, the use of altmetrics lead to some potential disadvantages, especially when they are used as the only indicator for impact assessment. There is also the difficulty with field normalization, which makes it difficult to compare the impact of different disciplines [78]. Besides, altmetrics could be affected by an incomplete and biased coverage of impact areas (eg, most Chinese regions do not use Twitter), which makes it difficult to compare the impact of different regions [79]. Importantly, altmetrics present a lack of quality control, and as such they are susceptible to deliberate or accidental manipulation, which may promote sensational outcomes, and the subsequent loss of credibility, if they are used as a sole indicator for impact assessment [80]. However, some of these drawbacks can be controlled when analyzing a large set of documents, and in this sense alternative metrics seem to be more prevalent and useful in health sciences when compared with other fields [81,82]. Thus, we consider that new alternative metrics are not replacing the classical ones. Indeed, these are 2 different approaches with a common goal: traditional metrics attempt to assess the scholarly impact among researchers, whereas alternative metrics try to evaluate policy and societal demands on a specific scientific issue. Although these approaches are different, they are positively correlated [26]. A randomized controlled trial [83] reported a causal relationship between the dissemination of research results through a web-based platform and subsequent citations.

Another limitation of this study is that we have restricted our search strategy to WoS, without exploring the presence of TE in other databases such as Scopus or Medline or employing a broader search strategy as reported in other studies [84]. However, WoS covers more than 250 scientific disciplines and its total number of records is over 90 million [85]. When performing bibliometric analysis, citation data provided by WoS are considered one of its main advantages in comparison with other databases. Furthermore, the coverage of TE documents is not limited by the date of WoS construction (1960s), as the seminal paper on TE was published in 1993 [1].

### Comparison With Prior Work

Previous studies of our group have described the global trends [10] and identified the cognitive and social framework of TE [11]. However, to our knowledge, this is the first study analyzing the online social dimension of TE as a research field in the age of mass media.

### Summary of Findings

1. Online social media play a key role in the dissemination of information about advanced therapies and TE from academics to patients and health consumers.
2. The focus of TE research groups at the academic level and the most shared articles in the online mass media are not the same, as the ranking of the top 10 most cited TE documents in terms of normalized WoS citations and normalized Altmetric Attention Score were not homogeneous.
3. Mathematical models established based on information retrieved from alternative metrics (altmetrics) can be used to predict the impact of TE documents on citation counts.
4. Different actors (academics, groups of basic and translational researchers, health clinicians, data managers, and health information workers) should implement knowledge diffusion models about advanced therapies and TE in the online mass media.

### Conclusions

TE has supposed a revolution in daily medical practice as tissue constructs are now available to treat severe conditions that previously remained untreated. Therefore, these new medical approaches have an impact on the population that can now be measured by altmetrics. These metrics differ from the classical academic metrics, but the knowledge of their influence on the final citation count could form the basis of different institutional or personal decision processes. The different actors involved in the scientific diffusion of the TE can use the results of this study to increase their interest in the use of social media and other online platforms as a window to the world, with the intention of reaching not only the scientific community, but also the general society.

## Abbreviations

TE: tissue engineering
WoS: Web of Science
